# Catatonia-like behavior and immune activation: a crosstalk between psychopathology and pathology in schizophrenia

**DOI:** 10.1186/s12991-023-00471-0

**Published:** 2023-10-11

**Authors:** Antonino Messina, Filippo Caraci, Eugenio Aguglia, Maria Salvina Signorelli

**Affiliations:** 1 Department of Clinical and Experimental Medicine, Psychiatry Unit, University of Catania, Catania, Italy; 2 Department of Drug and Health Sciences, University of Catania, Catania, Italy; 3 Neuropharmacology and Translational Neurosciences Research Unit, Oasi Research Institute-IRCCS, Troina, Italy

**Keywords:** Schizophrenia, Blunted affect, Flat affect, Psychomotor slowing, Catatonia, Catatonia-like behavior, Lymphocytes/monocytes ratio, Neutrophil/lymphocyte ratio

## Abstract

**Background:**

In Kalhbaum's first characterization of catatonia, the emotional symptoms, such as decreased or restricted expression of feelings and emotions, which is described as blunted affect, are related to the motor symptoms. In later years, the affective domain was excluded from the concept of catatonia and was not included among the diagnostic criteria in the various Diagnostic Statistical Manual (DSM) versions. In recent times, some authors have proposed the proposition of reevaluating the notion of catatonia through the reintroduction of the affective domain. The objective of this study was to examine the correlation between catatonic-like behavior (CLB), such as emotional withdrawal, blunted affect, and psychomotor slowing, and inflammatory markers, namely the neutrophil/lymphocytes ratio (NLR) and lymphocytes/monocytes ratio (LMR), in individuals diagnosed with schizophrenia.

**Method:**

A sample of 25 patients with schizophrenia (10 females, 15 males) was recruited, and the Brief Psychiatric Rating Scale (BPRS) was used to assess the severity of emotional withdrawal, blunted affect, and psychomotor slowing.

Findings: The correlation analysis (Spearman *ρ*) revealed a robust direct association between blunted affect and psychomotor slowing (*ρ* = 0.79, *P* = 0.001), and a significant direct correlation between CLB (emotional withdrawal, *ρ* = 0.51, *P* = 0.05; blunted affect *ρ* = 0.58, *P* = 0.05; motor retardation, *ρ* = 0.56, *P* = 0.05) and LMR (*ρ* = 0.53, *P* = 0.05).

In addition, patients with a duration of illness (DOI) older than five years had a higher presence of CLB and a higher LMR than patients with a more recent diagnosis of the disease. Likely, patients with positive symptoms and in the prodromal and active stages of the disease have a different immune profile than patients in the residual stage and with a predominance of negative symptoms.

**Conclusions:**

Psychomotor slowing and blunted affect are two significantly related features, representing the two-faced Janus of immobility. Furthermore, aggregating them in CLB is more predominant the longer the duration of schizophrenia and is associated with different a specific pattern of immune activation.

## Introduction

In 1911 Eugene Bleuler published “Dementia Praecox or the Group of Schizophrenias” [1]. The novelty with respect to Emil Kraepelin, who had previously identified dementia praecox, was that the deficit or negative symptoms of the disease were considered more relevant than delusions and hallucinations [2]. The negative symptoms Bleuler had identified in schizophrenia represented the cornerstones of the loosening association concept. Bleuler's concept of loosening association contained the core symptoms of schizophrenia, namely the “4 A’s” for association, affect, ambivalence, and autism [2]. In this way, the paradigm of schizophrenia described by Kraepelin, which favored delusions and hallucinations as core symptoms, was enriched with Bleuler's description.

Negative symptoms, which significantly impair functional outcomes and quality of life for patients with schizophrenia, include social withdrawal, reduced motivation and speech, blunted or flat affect, and psychomotor slowing [3].

Even though blunted affect (reduced gestures, facial and vocal expression, poor eye contact) [4] and psychomotor slowing have been studied and considered separately, however, some data in the literature, based on neuroimaging studies, point to the close connection between emotions and psychomotor activity [5]. About 50 percent of patients with schizophrenia show psychomotor slowing, and it has been observed that patients with schizophrenia who exhibit more significant psychomotor slowing have worse social functioning, poorer quality of life, and lower life expectancy [6]. In view of the importance of psychomotor slowing, unrelated to antipsychotics, in schizophrenia, some authors reputed it as “the closest thing to a north-star in schizophrenia research” [7]. In fact, motor control is not simply an activity in which only the frontal motor cortex is involved, but rather represents a complex and integrated activity in which several brain areas, such as prefrontal and parietal cortex, basal ganglia, cerebellum cooperate so that the motor act can be performed [8].

Firstly, Karl Ludwig Kahlbaum, in 1874, had identified and described psychomotor slowing as a pivotal symptom of psychosis [9]. Interestingly, Wernicke distinguished intrapsychic akinesia, which is related more to spontaneous movements and connected to motivation, from motor akinesia, which represented more reactive and externally stimulated motor acts [10]. The extreme form of psychomotor slowing, described by Kahlbaum and associated with waxy flexibility, akinesia, posturing, rigidity, repetitive speech/acts, and mutism to stupor, was identified as catatonia [9].

The basic symptoms of catatonia can be classified into different domains [11, 12]:

1. Motor signs: psychomotor slowing, waxy flexibility, immobility, mutism, stupor.
2. Behavioral signs: negativism, agitation.
3. Autonomic instability: tachycardia, hyperthermia, diaphoresis (Stauder's Malignant Catatonia) [13].
4. Inability to suppress motor functions: stereotypy, echolalia, echopraxia.

Besides the classic form of catatonia, described by Kahlbaum and taken up by the Diagnostic and Statistical Manual of Mental Disorders (DSM), Wang and coll. have described a clinical entity named catatonia-like behavior (CLB), in which the signs of psychomotor slowing, blunted affect, fluctuating mutism, social inhibition, are coexisting [14]. Catatonia, which in earlier versions of the DSM was considered a specification of schizophrenia, has become an independent clinical entity in DSM-5 [15].

In this regard, a neuroinflammatory state has been recognized as a causal factor in some forms of catatonia [16]. Limbic encephalitis, associated with antibodies against the N-methyl-D-aspartate receptor (NMDAR), often presents with typical symptoms of catatonia, such as mutism, stereotyped movements, negativism, and psychomotor slowing [17]. The immune alterations and activation of microglia observed in catatonia and schizophrenia could underlie the same pathogenetic process represented by neuroinflammation [16–19].

Therefore, a minimally invasive and inexpensive, yet reliable and useful modality to study the inflammatory status of patients with schizophrenia is the neutrophil-to-lymphocyte ratio (NLR) and lymphocyte-to-monocyte ratio (LMR) quantification. Several data have reported that the dosage of NLR and LMR was altered in schizophrenia and associated with the severity of the disease [20–24]. NLR and LMR, far from being exclusively markers of inflammation, are also indices of different immune system activation [24]. Variability in NLR depends on both stage of schizophrenia and treatment with antipsychotics [20]. To our knowledge, no studies have explored the relationship between CLB (blunted affect, emotional withdrawal and psychomotor slowing) and inflammatory markers (NLR and LMR) in individuals diagnosed with schizophrenia. This preliminary study investigates the association between blunted affect, psychomotor slowing, and inflammatory markers.

## Materials and methods

### Participants

A retrospective analysis, aimed to investigate the relationship between immune activation and CLB, was conducted on a sample of 25 randomized clinical records of outpatients diagnosed with schizophrenia (DSM-5-TR), with an illness duration of less than 10 years. The sample consisted of 10 female and 15 male individuals, aged between 22 and 50 years. The clinical records were obtained from the University Psychiatric Unit located in Catania, Italy. The inclusion criteria for the subjects were a confirmed diagnosis of schizophrenia according to the DSM-5-TR [25] criteria and the ability to provide informed consent. Patients with diagnosed substance abuse or cognitive disorders in comorbidity were excluded from the study. Patients with previous or current use of D2 receptor antagonist (e.g., haloperidol, amisulpride) were also excluded. Individuals who met the inclusion criteria were approached by the research team and provided with detailed information about the study. Informed consent had been obtained from all participants before using their clinical data. The participant IDs were assigned for identification purposes and did not represent any specific information about the individuals. The data were gathered and stored within a database.

### Clinician administered rating scale

All patients have been previously evaluated, during a routine assessment, for negative symptoms through the Brief Psychiatric Rating Scale (BPRS) [26] by a specialist adequately trained in using the rating scale. This rating scale consists of 18 items, and it was specifically designed to screen various trans-nosographic domains belonging to different psychiatric disorders, such as affectivity disorders, anxiety disorders, and psychotic disorders. Each item of the BPRS is rated on a scale from 0 to 7, with higher scores indicating greater severity. A meta-analysis was conducted to examine factor analyses of the BPRS using the specified parameters. The confirmation of the validity of the four subscales was achieved by an analysis of the 12 individual items that constituted those subscales. The findings of the meta-analysis point to the existence of five subscales: affect (anxiety, guilt, melancholy, and physical symptoms); positive symptoms (grandiose thinking, hallucinogenic behavior, and conceptual disarray); negative symptoms (blunted affect, emotional withdrawal, and motor slowing) [27].

Some specific items provide a comprehensive and reliable measure of the negative symptomatology. In this regard, for this study aimed at identifying and quantifying CLB, three items were used:

- Item 3: Emotional withdrawal
- Item 13: Motor retardation (psychomotor slowing)
- Item 16: Blunted affect.

Trained researchers have administered the BPRS to all participants individually. The BPRS had been administered standardized, following the guidelines provided by the scale developers. Care was taken to maintain a supportive and non-threatening environment during the administration process. The psychometric evaluation had been retrieved from medical records.

### Blood sample collection

The blood sample had been obtained, during routine evaluation, with a butterfly needle and collected inside a tube with EDTA from all patients at the same hour (8:30 a.m.). Within three hours after collection, the blood had been centrifuged. The absolute value of neutrophils, lymphocytes, and monocytes was considered in order to evaluate the absolute ratio of neutrophils to lymphocytes and lymphocytes to monocytes. Blood count report data had been extracted from the medical records.

### Data acquisition and statistical analysis

After completing the BPRS and calculating the NLR and LMR, the data were collected and recorded for each participant. The scores of the three specific CLB items obtained from the BPRS, the NLR, and the LMR were entered into the SPSS statistical analysis software 25 version for further analysis. Statistical tests, such as Spearman correlation analysis, were calculated to verify the relation between blunted affect, psychomotor retardation, emotional withdrawal, NLR, and LMR. Means were compared with the non-parametric *U*-Mann–Whitney test. For all statistical analyses, a *P*-value of < 0.05 was considered significant.

## Results

The demographic data and results were collected and presented in the table below (Table 1). No significant difference was observed between the sexes and between smokers versus nonsmokers in relation to psychic domains (emotional withdrawal, blunted affect, and psychomotor slowing) and inflammatory markers (NLR and LMR) (*P* ≥ 0.05).

**Table 1:** Demographic data and results of catatonia-like behavior items and markers of inflammation

Age	39.6 (5.6)
Males	15 (53.8%) total (*n *= 25)
Duration of illness	5.9 (2.2)
Education	10 (1.3)
Smokers	13
NLR	3.3 (1.8)
LMR	14.1 (2.9)
Emotional withdrawal	4.3 (1.2)
Blunted affect	5.8 (1.5)
Psychomotor slowing	4.9 (1.4)

The correlation coefficient of Spearman (*ρ*) indicated a relevant positive correlation between blunted affect and psychomotor slowing (*ρ* = 0.79, *P* = 0.00). This finding suggests that as the severity of blunted affect increases, there is a tendency for motor retardation also to increase. A similar positive correlation was found between emotional withdrawal and psychomotor slowing. (*ρ* = 0.71, *P* 0.01). The positive correlation implies that these two affect domains tend to occur together with psychomotor slowing. A significant direct correlation was observed between CLB and LMR (*ρ* = 0.53, *P* = 0.05). Separating each dimension from the concept of CLB yields the following correlations with LMR: emotional withdrawal, *ρ* = 0.51, *P* = 0.05; blunted affect *ρ* = 0.58, *P *= 0.05; psychomotor slowing, *ρ* = 0.56, *P* = 0.05. On the contrary, the same significance was not achieved with respect to the NLR. Moreover, controlling for the duration of illness by partial correlation, the significance of the correlation between CLB and LMR was not missed.

Statistical analysis revealed that patients with more than 5 years of disease had a significantly higher LMR (Mann–Whitney *U* = 12.5, *P* = 0.001) (Table 2). The same cannot be said regarding the NLR, which tended to appear higher in patients with less than 5 years of disease without, however, reaching significance (Table 2). Furthermore, by aggregating the resulting scores for the three items of CLB and comparing with respect to duration of illness, we observed a higher occurrence of CLB among patients with a DOI greater than 5 years (Mann–Whitney *U* = 21.0, *P* = 0.009) (Table 2).

**Table 2:** Comparison of markers of inflammation and CLB^a^ versus DOI^b^

	DOI^b^ < 5 years	DOI^b^ > 5 years
LMR^c^	11.8 (0.7)*	13.1 (0.8)*
NLR^d^	3.6 (2.1)	3.2 (1.1)
CLB^a^	9.0 (2.6)*	12.4 (2.5)*

*Data are expressed as mean (SD)*

^*^Significance *P* < 0.05

^a^*CLB*: catatonia-like behavior

^b^*DOI* duration of illness

^c^*LMR* lymphocyte/monocyte ratio

^d^*NLR* neutrophil/lymphocyte ratio

## Discussion

This preliminary study highlighted, above all, the strong association between blunted affect and psychomotor slowing. This finding is consistent with the concept of catatonia formulated by Kahlbaum, according to whom catatonia included not only motor disorders (the only ones to date transposed by the DSM-5-TR [25]), but also blunted affect and psychomotor slowing [9, 28–31].

In a review, Hirjac and Coll. [30] delve into the historical, neuroanatomical neurotransmitter, and clinical reasons for considering blunted affect and psychomotor slowing as closely related and part of a unique syndromic pattern.

Disrupted connections of intercortical (“horizontal modulation”) and subcortical–cortical (“vertical modulation”) circuits between the orbitofrontal, prefrontal (dorsolateral and ventromedial, parietal cortex, and basal ganglia found in catatonia, are the common neuroanatomical basis between affective and psychomotor disturbances [32–35]. Thus, different brain areas engaged in emotion and sensorimotor processing operate synergistically in elaborating the motor program and execution [35, 36]. In addition, cognitive domains, such as attentional deficits and impaired motor planning, could contribute to blunted affect and psychomotor slowing [36]. In this regard, the mirror neuron system that represents a neural substrate, activated when an action or emotion is expressed and observed [37, 38], plays a pivotal role in emotional processing and motor execution. A recent review showed that mirror neurons are distributed in multiple brain networks, which encompass the same areas involved in catatonia, such as the prefrontal cortex, parietal cortex, amygdala, cerebellum, and basal ganglia. Mirror neurons also contribute to high mental functions and are crucial in motor, perceptual, emotional, and behavioral tasks [39]. It has been observed that drug-naive patients with schizophrenia exhibit a dysfunction in the neuron mirror system concerning patients with schizophrenia taking antipsychotics [40, 41]. Therefore, the psycho (affective and cognitive) and the motor components are closely related [36], and blunted affect, and psychomotor slowing represents a two-faced Janus of immobility. From an evolutionary point of view, “tonic immobility” represents a primordial mode of defense against prey, in which motor freezing could have been helpful in defense against predators [42].

The other aspect regards neurotransmission abnormalities observed in schizophrenia as in catatonia. Dopaminergic alterations account for psychomotor retardation, while GABAergic and glutamatergic alterations are responsible for blunted affect. Therefore, CLB, catatonia, and schizophrenia share a unique pathogenetic process that implies the cortical glutamatergic/GABAergic and the dopaminergic cortico-subcortical modifications [30].

**Fig. 1:**
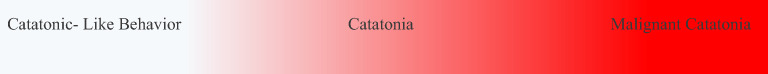
The spectrum of the catatonia

Although catatonia is present in 10–25% of patients with schizophrenia [43], if we consider CLB as part of the spectrum of catatonia, it may be underestimated. The prevalence of CLB in the setting of patients with schizophrenia could be considerably higher. Because of the neurobiological concordance between schizophrenia and CLB, as well as historical, it could be recognized as a first-rank symptom. Thus, it seems appropriate that all patients with schizophrenia should be screened for catatonia symptoms, in order to identify subthreshold symptoms of catatonia (CLB) with minimal impact on motor and affective function, from the most severe cases of catatonia, including motor block, negativism, and mutism to stupor and malignant catatonia (Fig. 1).

The CLB can significantly impact daily functioning, social ability and contribute to disability in patients with schizophrenia and poor prognosis [44]. Given that the circuits involved in the pathogenesis of schizophrenia would overlap with those that preside over motor activity, psychomotor slowing could represent a valid endophenotype for schizophrenia [45]. The clinical finding in our study finds further confirmation in a paper published by Cohen and Coll. [46] who, based on the Research Domain Criteria (RDoC) concept developed by the National Institute of Mental Health (NIMH), proposed to include blunted/flat affect, alogia, and psychomotor slowing within a single trans-nosographic category termed “reduction in expressive behaviors”.

However, it should be observed that the use of D2 receptor antagonists (e.g., butyrophenones, phenothiazines) frequently induces the appearance of blunted affect and psychomotor slowing due to the strong antagonism on nigrostriatal D2 receptors. However, introduction of D2 and 5-HT2 receptor antagonists has dramatically reduced the deleterious action on affectivity and motor. Moreover, psychomotor changes appear independent of drug therapy, even in drug-naive patients [47].

**Fig. 2:**
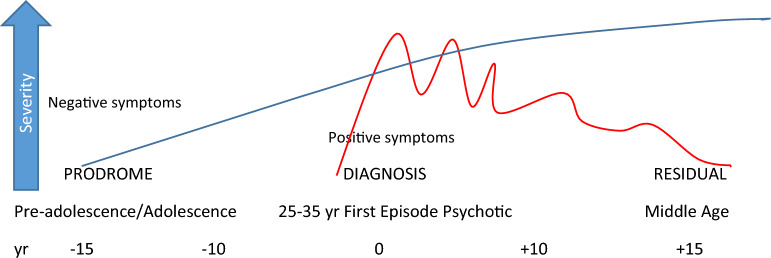
Schizophrenia’s course

During schizophrenia, negative symptomatology progresses and worsens over the years of illness until the terminal residual phase [4]. While positive symptoms (e.g., delusions and hallucinations) prevail in the active phase, later in the long-term positive symptoms decline, and negative symptoms become more prominent [4] (Fig. 2).

Our study observed that patients with schizophrenia with a DOI greater than 5 years had a higher presence of CLB. In addition, higher DOI and the presence of CLB were associated with a significantly higher LMR compared with patients with lower DOI than 5 and lower CLB. It is conceivable that each phase of schizophrenia is theoretically related to a different activation of the immune system. In contrast, NLR did not differ from DOI and did not correlate with CLB.

Likely in the prodromal and active phases of schizophrenia, an activation of the innate immune response predominates with greater activation of neutrophils, while in the residual phases, when negative symptoms are.

Likely in the prodromal and active phases of schizophrenia, an activation of the innate immune response predominates with greater activation of neutrophils, while in the residual phases, when negative symptoms are prevalent, activation of lymphocytes, typical of the adaptive immune response, would be prevalent. Following what has been reported in our study, it was observed that a high blood concentration of Interleukin-17 (IL-17), which stimulates neutrophil proliferation [48], was found in first-episode psychotic patients [49].

Lymphocytes, on the other hand, are downregulated during neutrophil activation (as has been shown in acute infectious forms, e.g., COVID-19) [50], and this may explain the high LMR in the residual stages of schizophrenia, compared with the early stages of the disease.

The findings of this preliminary study have clinical implications for the assessment and treatment of individuals with schizophrenia. Clinicians should consider assessing both blunted affect and psychomotor slowing as part of a comprehensive evaluation. Based on the results of a meta-analysis conducted by Shafer, the BPRS is used to identify negative symptoms [27] and provide a specific assessment of catatonic features within the overall psychopathology framework.

Recognizing the association between CLB and inflammation markers can aid clinicians in evaluating, monitoring, and staging the symptomatology of patients. CLB can occur at the onset of schizophrenia, and their assessment and treatment overall functioning, quality of life and outcome [51, 52]. Early detection and prompt treatment of catatonic symptoms is critical to avoid chronicity [53].

Moreover, individuals diagnosed with schizophrenia have a decrease in their overall life expectancy due to the presence of comorbidities associated with internal medical conditions. Given that catatonia can be linked to a range of organic diseases, it is mandatory that a comprehensive medical evaluation be conducted in all patients displaying symptoms related to catatonia, as well as in patients with psychiatric disorders in general (Table 3) [54].

**Table 3:** Non-psychiatric causes of catatonia

Electrolyte imbalances
Hepatic encephalopathy
Uremia
Endocrine disorders (thyroid disorders, Addison's disease, parathyroid disorders)
Autoimmune disorders (systemic lupus erythematosus, anti-NMDA receptor encephalitis)
Infections (encephalitis, neurosyphilis)
Neurological conditions (Parkinson disease, cerebrovascular disease, multiple sclerosis, brain tumors)
Others (drugs, B12 vitamin and folic acid deficiencies, brain hypoxia)

Several organic conditions can also cause or precipitate catatonia. Recognizing these is critical because treatment and prognosis can differ depending on the underlying cause. Catatonia can be a severe condition, and if not treated, it can lead to complications such as rhabdomyolysis, pressure ulcers, aspiration pneumonia, renal failure, hyperthermia, dehydration, and deep vein thrombosis [55].

The investigation into the involvement of inflammation in schizophrenia and the possible therapeutic benefits of anti-inflammatory drugs has garnered increasing attention. Minocycline is a pharmacological agent with antibacterial characteristics and the ability to reduce inflammation. Several clinical investigations have shown that the adjunctive use of minocycline with antipsychotic therapies may enhance symptomatology in individuals with schizophrenia [56]. Six randomized controlled trials (RCTs) were selected for inclusion in a review. The conducted trials have yielded data that support the superiority of minocycline over placebo, demonstrating its effectiveness in ameliorating negative symptoms associated with schizophrenia [57]. Furthermore, anecdotal studies have suggested that the administration of minocycline has shown promise in ameliorating the symptoms of catatonia, particularly in individuals diagnosed with schizophrenia [58, 59]. Nevertheless, conducting more randomized controlled trials (RCTs) that adhere to rigorous methodological standards and encompass bigger sample sizes is necessary to validate the findings.

Thus, psychosocial interventions, cognitive remediation, clinical and pharmacological approaches, purposeful to treat the negative symptoms and underlying inflammation could help to find an answer to unmet needs still in the treatment of schizophrenia [7, 56].

### Limitations

The current study has several limitations. First of all, the small sample number is a critical issue. In addition, more homogeneous groups should be established with regard to disease stages so that we can subdivide the sample into onset psychosis, active phase psychosis, and residual psychosis. Recruit a diverse group of participants in terms of age, gender, ethnicity, and underlying conditions, since catatonia can present in various conditions, including mood disorders, schizophrenia, and general medical conditions. It would be appropriate to consider the type of treatment the patients were on, thus differentiating drug-naive patients from patients taking first- or second-generation antipsychotics and patients taking two antipsychotics. In future investigations, it would also be desirable to use more specific rating scales to assess catatonia and CLB. Identifying predictors of CLB in schizophrenia might be accomplished by undertaking regression analyses or, in large-scale research, by adopting machine-learning and deep-learning techniques. These methods can potentially validate the function of markers such as LMR, NLR, CRP, and cytokines, among others, in the etiology of CLB in schizophrenia. In add, use a combination of clinical assessments, neuropsychological tests, neuroimaging, and other techniques to get a comprehensive understanding of catatonia. Other objective measures, such as neuroimaging or neurophysiological assessments, could provide complete and reliable data on CLB and inflammatory markers. Since catatonia can have varying courses and outcomes, consider a longitudinal study design to track changes and outcomes over time and recognize and control for other medical or psychiatric conditions that might be present alongside catatonia.

Finally, the research focused solely on the correlation between emotional withdrawal, blunted affect, and psychomotor slowing. It did not investigate other potential factors (e.g., positive symptoms) or variables that may influence this relationship.

## Conclusion

Schizophrenia should be framed as a systemic disease in which inflammation and immune alterations are the pathological humus on which the brambles of neural networks associated with psychopathology develop.

The conclusion we can draw from this preliminary study can be summarized in these points:

- Blunted affect, emotional withdrawal and psychomotor slowing appear closely related to each other, justifying including them in a unique framework well delineated by the CLB.
- CLB-related symptoms (emotional withdrawal, blunt affect, and psychomotor slowing) are significantly correlated with LMR.
- Patients with DOI > 5 years have a higher predominance of CLB than patients diagnosed with more recent schizophrenia.
- Patients with more than 5 years of disease have a pattern of immune activation related more to lymphocytes and adaptive immunity (higher LMR than patients with DOI < 5 years, who tend to have a prevalence of neutrophils, involved in innate immunity).
- Screening all patients with schizophrenia for CLB and investigating inflammatory markers could individualize treatment and ultimately improve prognosis.

## Data Availability

Data used to generate the results in this study are available for non-commercial purposes upon request to the lead author.
